# Application of the “3-2-1” body surface localization method in intertrochanteric femoral fractures: a technical note

**DOI:** 10.3389/fsurg.2024.1394575

**Published:** 2024-08-29

**Authors:** Xiaowei Wu, Yanbin Lin, Yangkai Xu, Linglan Yan, Shaochen Tu

**Affiliations:** Department of Orthopedics, Fuzhou Second Hospital, Fuzhou, China

**Keywords:** intertrochanteric femoral fracture, incision location, surgical procedure, technical note, innovative device

## Abstract

In femoral intertrochanteric fractures, poor incision positioning may result in inaccurate intramedullary nail placement direction, which increases the difficulty of reduction and thus the size and number of incisions. Repeated intraoperative fluoroscopy not only increases the radiation exposure of the surgeon but also affects the operative outcomes. This technical note proposes a method of identifying incision positioning preoperatively using the “3-2-1” body surface localization method. This auxiliary positioning technique uses a body surface locator and the lower limb force axis. It can predict the incisions for the needle insertion point, spiral blade, and locking nails, create minimally invasive incisions, avoid incorrect incision position, facilitate accurate intraoperative intramedullary nail placement, reduce the incision size, intraoperative bleeding, and radiation exposure, and improve surgical efficiency and reduction quality.

## 1 Introduction

Intertrochanteric femoral fractures are common hip fractures in older people, accounting for 46.5% of all hip fractures (1). The current mainstream fixation method is the proximal femoral nail antirotation (PFNA) fixation (2–4); compared with extramedullary fixation, PFNA fixation, which is associated with less soft tissue damage, less bleeding, and minimally invasive incision, is beneficial for fracture healing and improves patients’ postoperative quality of life. However, the traction of the abductor and rotator muscles around the trochanter of the femur resulted in the abduction, external rotation, and flexion of the proximal fracture mass, whereas the traction of the adductor muscles resulted in the inversion and shortening of the distal fracture mass (5). Reducing the fracture mass through minimally invasive incisions with intramedullary nails is challenging. Consequently, preoperative incision positioning planning is particularly important. Currently, preoperative positioning methods include robot navigation positioning (6, 7), guide pin fluoroscopy positioning (8), foraminal mirror grid positioning (9), and three-finger positioning (10); however, they all have shortcomings.

In this study, we describe the “3-2-1” body surface localization method, which effectively solves the shortcomings of previous methods such as high intraoperative equipment cost, inability to locate the position of the spiral blade incision, and individual differences. The intraoperative use of this positioning method avoids incorrect incision positioning, facilitates accurate intraoperative intramedullary nail placement, and reduces the incision size, intraoperative bleeding, and radiation exposure. Thus, the operation efficiency and reduction quality were greatly improved.

## 2 Methods

Currently, the supine position is generally selected in the treatment of older intertrochanteric fractures with PFNA because it is considered easy to execute and convenient for intraoperative fluoroscopy and facilitates evaluation. A few surgeons also use lateral position, believing that this position is convenient for nail insertion and reduction of other complex fractures and avoids complications caused by overtraction (11–13). Combining the advantages of both positions, the “3-2-1” body surface localization method reduces the operation time and intraoperative bleeding and promotes operation efficiency; thus, in this localization method, the supine position was used to facilitate position placement and intraoperative fluoroscopy.

**Figure 1:**
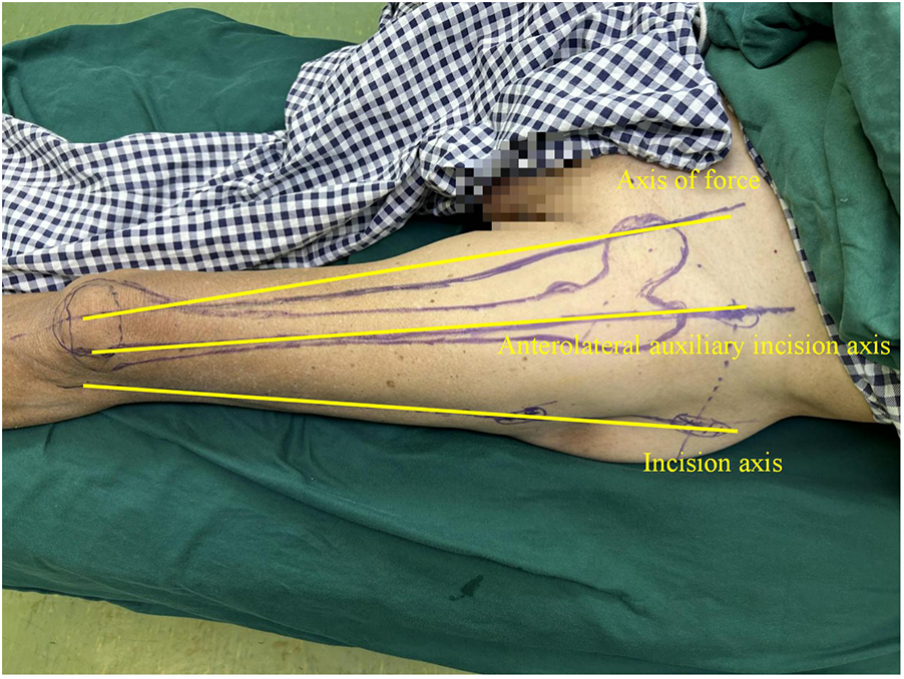
The surgeon uses a marker to draw three vertical axes on the patient's body surface. (1) Axis of force: 1/3 of the inguinal ligament is connected to the midpoint of the patella; (2) Anterolateral auxiliary incision axis: the normal anterior superior iliac spine is connected to the lateral edge of the patella; (3) Incision axis: side square center line of femur.

**Figure 2:**
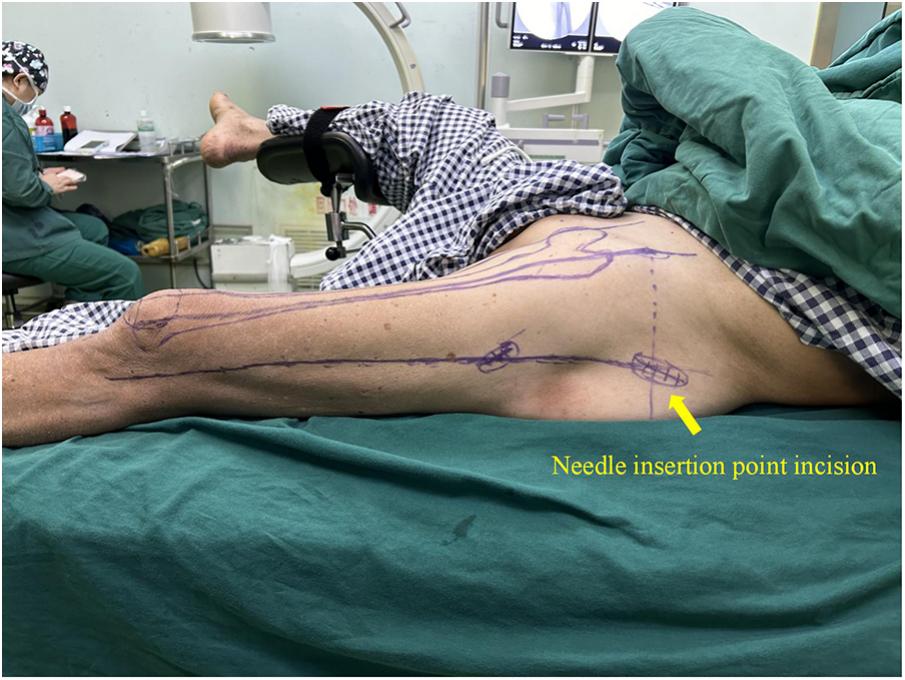
Needle insertion point incision: take the intersection of the vertical line of the anterior superior iliac spine and the midline of the thigh side as the center, and draw a marked line with a length of 3 cm slightly tilted back 15°.

**Figure 3:**
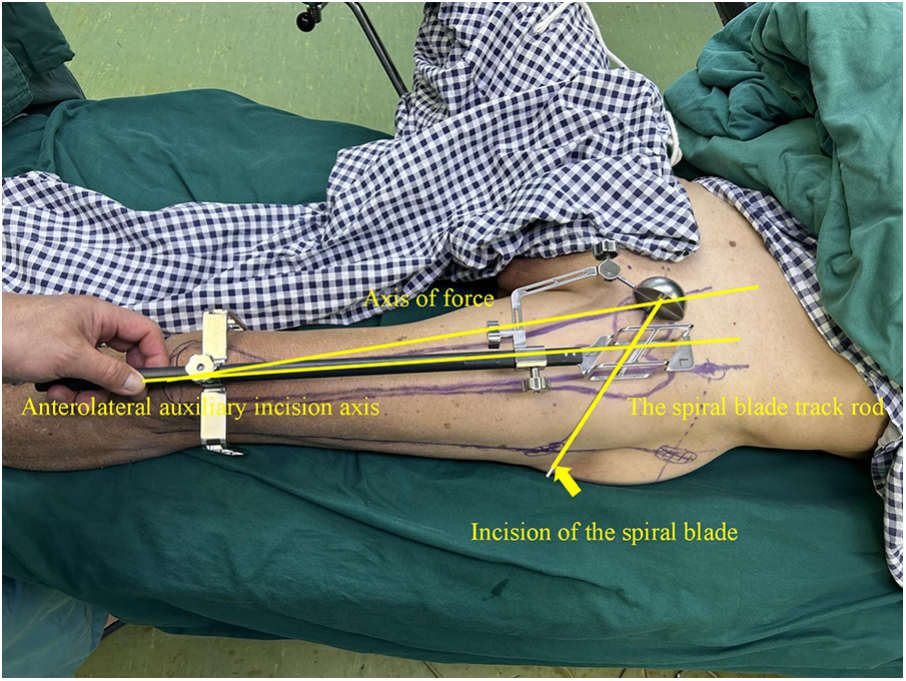
Incision of the spiral blade: marking the trajectory line of the spiral blade on the body surface through the body surface locator, and tilt it 15° to the head and neck with the intersection point of the midline on the thigh side as the center point, and the length is about 2 cm.

**Figure 4:**
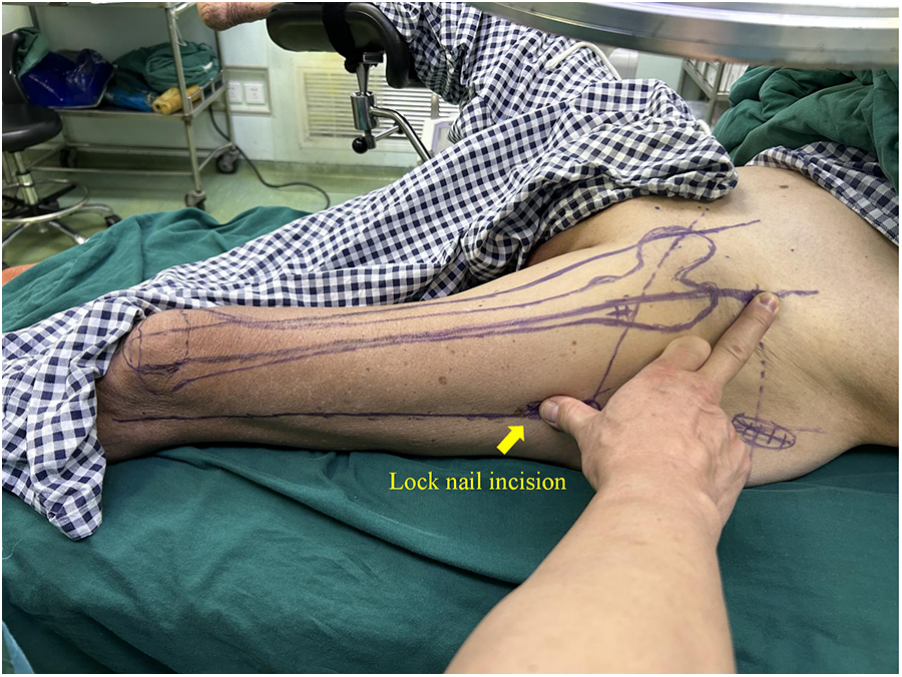
Incision of the spiral blade: an oblique inward and downward 1 cm incision was made at the median square line between the anterior superior spine of the iliac and the side of the femur, with the thumb and middle finger opening as the length.

**Figure 5:**
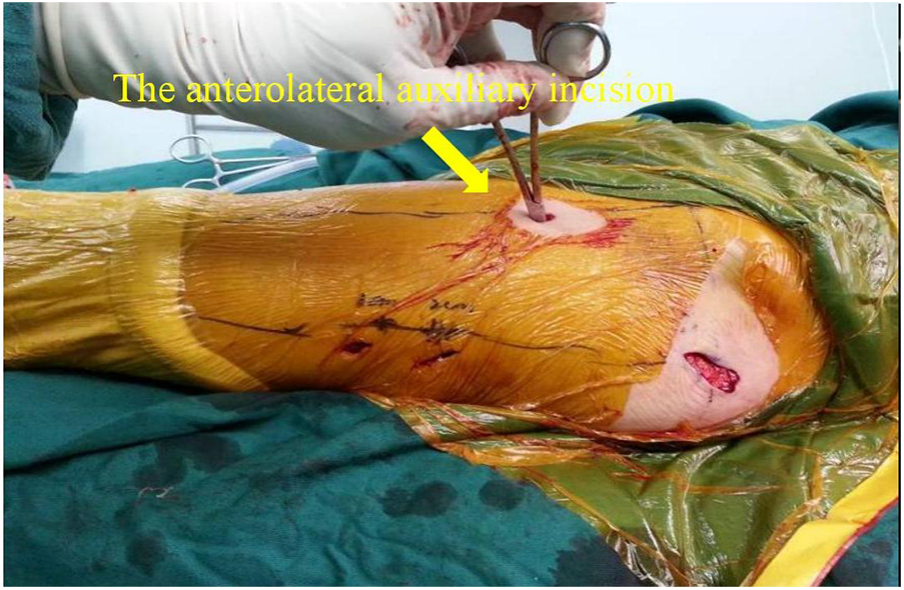
For fracture blocks that are difficult to reduce, an incision about 1 cm long can be made near the axis of the anterolateral auxiliary incision and the reference fracture position, and the reduction can be assisted by bending forceps.

**Figure 6:**
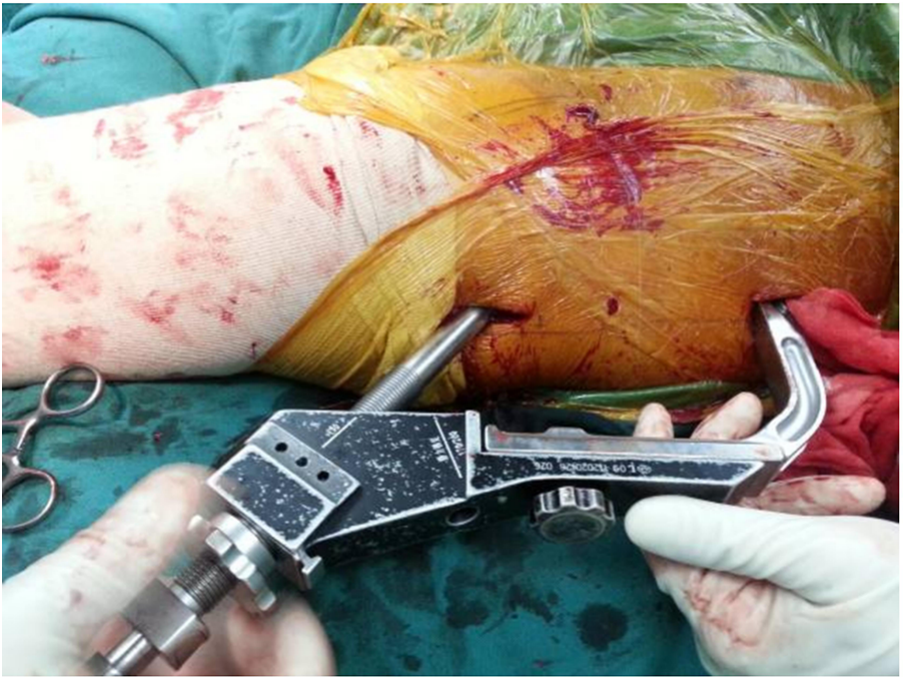
After the PFNA locator is installed, the spiral blade is exactly opposite the spiral blade incision, and the spiral blade incision plays a “one incision dual use” role.

**Figure 7:**
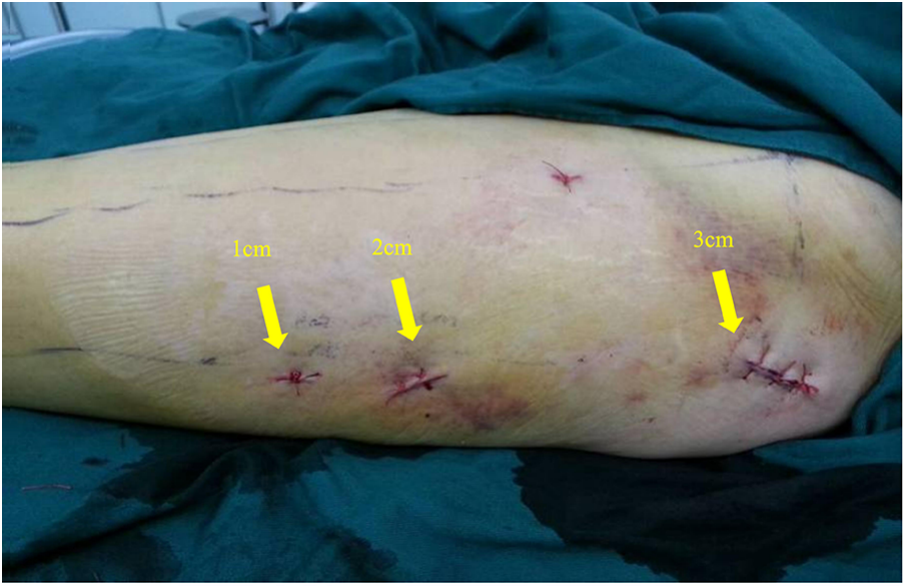
The postoperative incisions of the patients were minimally invasive, respectively “3 cm, 2 cm, 1cm”, hence the name “3-2-1”.

In this technique, the patient undergo surgical treatment on a single-leg lithotomy position while lying supine on a traction or a nontraction bed with “3-2-1” body surface localization before anesthesia induction. The “3-2-1” body surface localization method stands for the three minimally invasive incisions: 3, 2, and 1 cm. In the neutral position of lower-limb traction before operation, three longitudinal axes were determined (Figure 1): (1) A*xis of force*. One-third of the inguinal ligament is connected to the midpoint of the patella. (2) *Anterolateral auxiliary incision axis*: The normal anterior superior iliac spine is connected to the lateral edge of the patella. (3) *Incision axis*: This refers to the side square center line of the femur. Then, three minimally invasive incisions are positioned as follows: (1) *Needle insertion point incision* (Figure 2). A 3-cm marker line is drawn at the intersection of the vertical line of the anterior superior iliac spine and the midline of the thigh side, with the center slightly tilted 15° back, and the incision can be made according to the line. (2) *Incision of the spiral blade* (Figure 3). The trajectory line of the spiral blade is marked on the body surface through the body surface locator and tilted 15° to the head and neck with the intersection point of the midline on the thigh side as the center point, and the length is approximately 2 cm. (3) *Lock nail incision* (Figure 4). An oblique inward and downward 1-cm incision is made at the median square line between the anterior superior spine of the iliac and the side of the femur, with the thumb and middle finger opening as the length. After the induction of general anesthesia, patients are routinely disinfected and covered with towels. Closed reduction is attempted for fractures with obvious displacement. For fractures with difficult reduction, an incision approximately 1 cm long can be made near the axis of the anterolateral auxiliary incision near the reference fracture location (Figure 5), assisted by bending forceps for reduction, or after incisions for the needle insertion point and spiral blade are made, the reduction is performed through two or three incisions. After reduction, the needle insertion point incision is opened according to the marked line, and the skin, subcutaneous tissue, and fascia are cut successively while protecting the gluteus media. If the closure reduction is not satisfactory or the reduction is lost during nail placement, the spiral blade incision is opened simultaneously, and the spiral blade and anterolateral auxiliary incisions are used for reduction with the aid of bending forceps and finger lifting. In the anteroposterior position, the PFNA insertion point is usually located at the apex or slightly outside of the greater trochanter. Moreover, the design of the 6° external declination angle of the main nail can well match the configuration of the marrow cavity. On the lateral film, whether the guide needle is located in the center of the marrow cavity and is not curved is unclear. At the location of the needle insertion point incision, the index finger touches the apex of the greater trochanter or is slightly outside as the needle insertion point, insert the guide needle under perspective, adjust the direction of the guide needle, expand the marrow cavity, and place the main nail. After the installation of the PFNA locator, the spiral blade is placed through the spiral blade incision (Figure 6). After confirming the fracture position, the distal lock nail is placed through the lock nail incision, and the tail cap is installed. The incision is then rinsed and closed by layer. Postoperatively, the fracture was completely reduced, and the incision was minimally invasive, achieving “3-2-1” (Figure 7).

## 3 Materials and equipment

**Figure 8:**
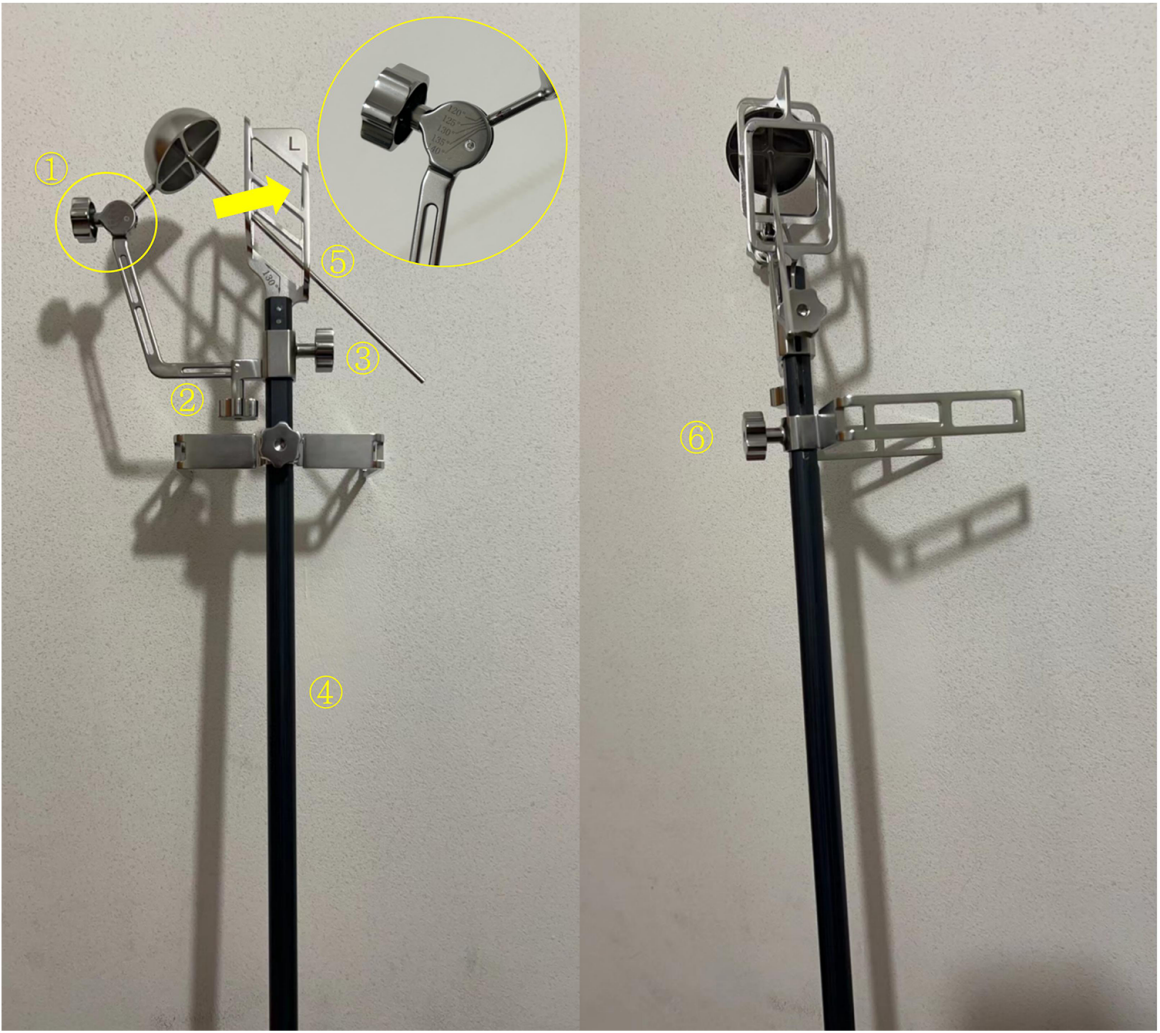
The above is the diagram of the body surface locator. “(1)” is the regulator with a range of 120–140 degrees, used to adjust the spiral blade track rod; “(2)” is an adjuster for moving the spiral blade track rod inside and outside; “(3)” is the adjuster for the spiral blade track rod to move up and down; “(4)” is the main rod of the body surface locator; “(5)” is the spiral blade track rod; “(6)” is the adjuster that moves the knee joint fixation device up and down.

**Figure 9:**
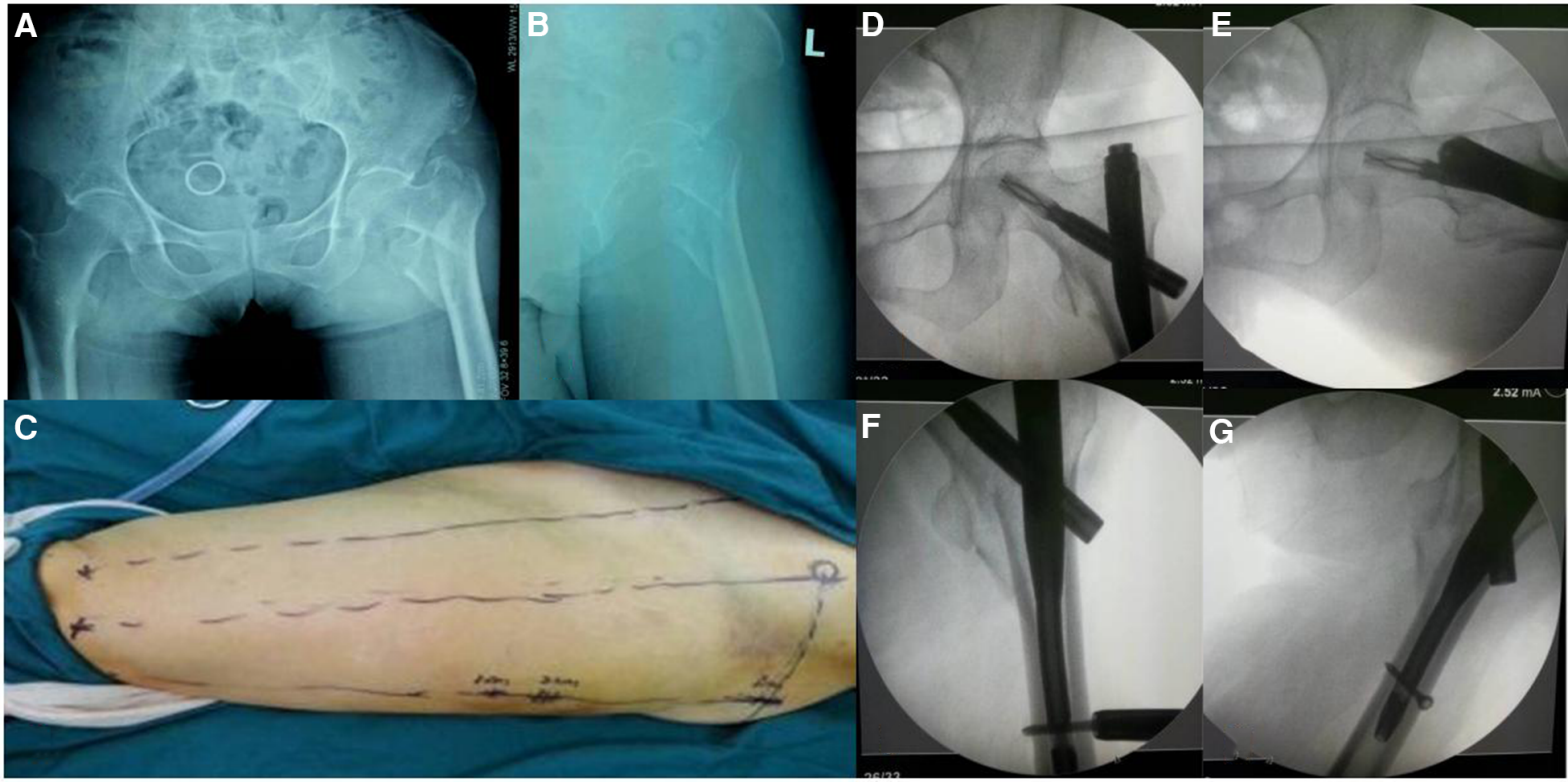
Preoperative anterolateral x-rays of the patient's hip joint **(A,B)** preoperative localization of skin incision was performed by the “3-2-1” body surface localization method **(C)** postoperative anterolateral x-rays of the patient's hip joint showed good fracture reduction **(D–G)**.

The body surface locator is composed of a main rod and a spiral blade track rod. The positioning in the “3-2-1” body surface localization method considers three vertical axes, namely, the axis of force, anterolateral auxiliary incision axis, and incision axis. When the body surface locator is used for positioning, the main rod corresponds to the anterolateral auxiliary incision axis. In Figure 8, “(6)” is adjusted to move down to the knee joint, and “2” and “3” are adjusted to correspond to the line between the acetabulum cup on the spiral blade track rod and the midpoint of the patella to the axis of force. Finally, under x-ray fluoroscopy guidance, the trajectory of the spiral blade is set to correspond to the axis of the femoral neck through the adjustment of “1” (which is adjusted to range from 120° to 140°), “2,” and “3.” By taking the intersection point of the spiral blade track rod and the midline of the thigh side as the center point, tilt 15° to the head and neck direction, and an incision of approximately 2 cm long is made for the spiral blade (Figure 9).

## 4 Case series

**Figure 10:**
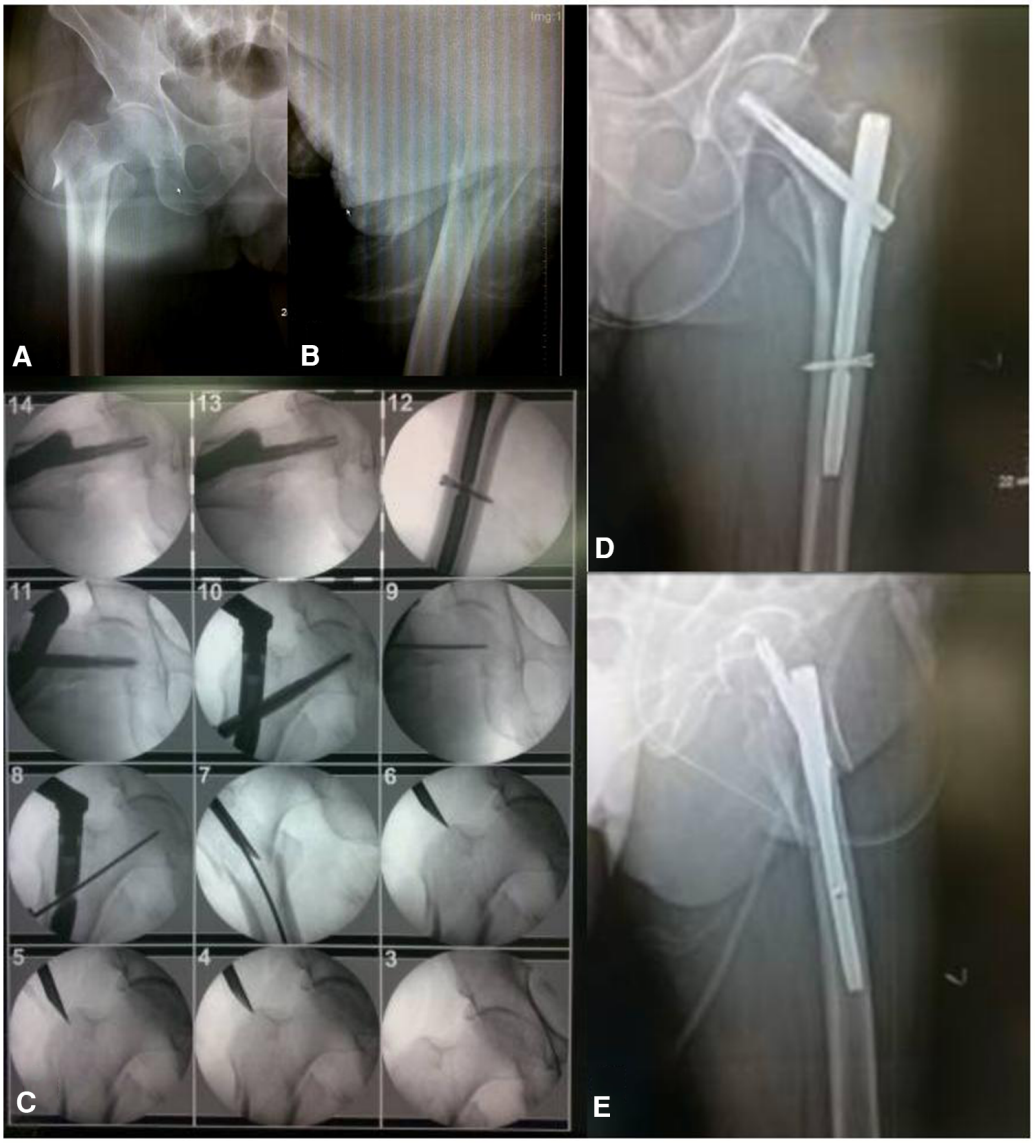
Preoperative anterolateral x-rays of the patient's hip joint **(A,B)** surgical procedure for PFNA **(C)** postoperative anterolateral x-rays of the patient's hip joint showed good fracture reduction **(D,E)**.

Case 1 referred to an older female patient who was admitted to the emergency department with a “left intertrochanteric fracture of the femur” that caused traumatic left hip swelling and pain (Figures 10A,B). The incision location was marked using the “3-2-1” body surface localization method (Figure 10C) before surgery, and PFNA was performed with a minimally invasive incision in the supine position under general anesthesia. Intraoperatively, the spiral blade and anterolateral auxiliary incisions were flexibly made for reduction with the help of flexure forceps and finger lifting of the lesser rotor, and the fracture was well reduced postoperatively (Figures 10D–G). The surgical incision was consistent with the preoperative plan, achieving truly minimally invasive surgery, greatly reducing the operation time, and significantly reducing intraoperative bleeding.

Case 2 referred to an 83-year-old male patient who was admitted to the emergency department for a “left intertrochanteric fracture,” (Figures 8A,B), traumatic left hip swelling, and pain. Preoperatively, the incision was marked using the “3-2-1” body surface localization method. PFNA was performed for the intertrochanteric femoral fracture under general anesthesia and in the supine position. The entire procedure took 15 min, excluding the time required for surgical sutures. With good fracture reduction, the operation time, intraoperative bleeding, and postoperative complications were greatly reduced (Figures 8C–E).

The “3-2-1” body surface localization method cannot only effectively hasten the operation time and reduce intraoperative exposure in the surgical treatment of intertrochanteric femoral fractures but also of multiple hip fractures (intertrochanteric fractures with subtrochanteric femoral and femoral neck fractures).

**Figure 11:**
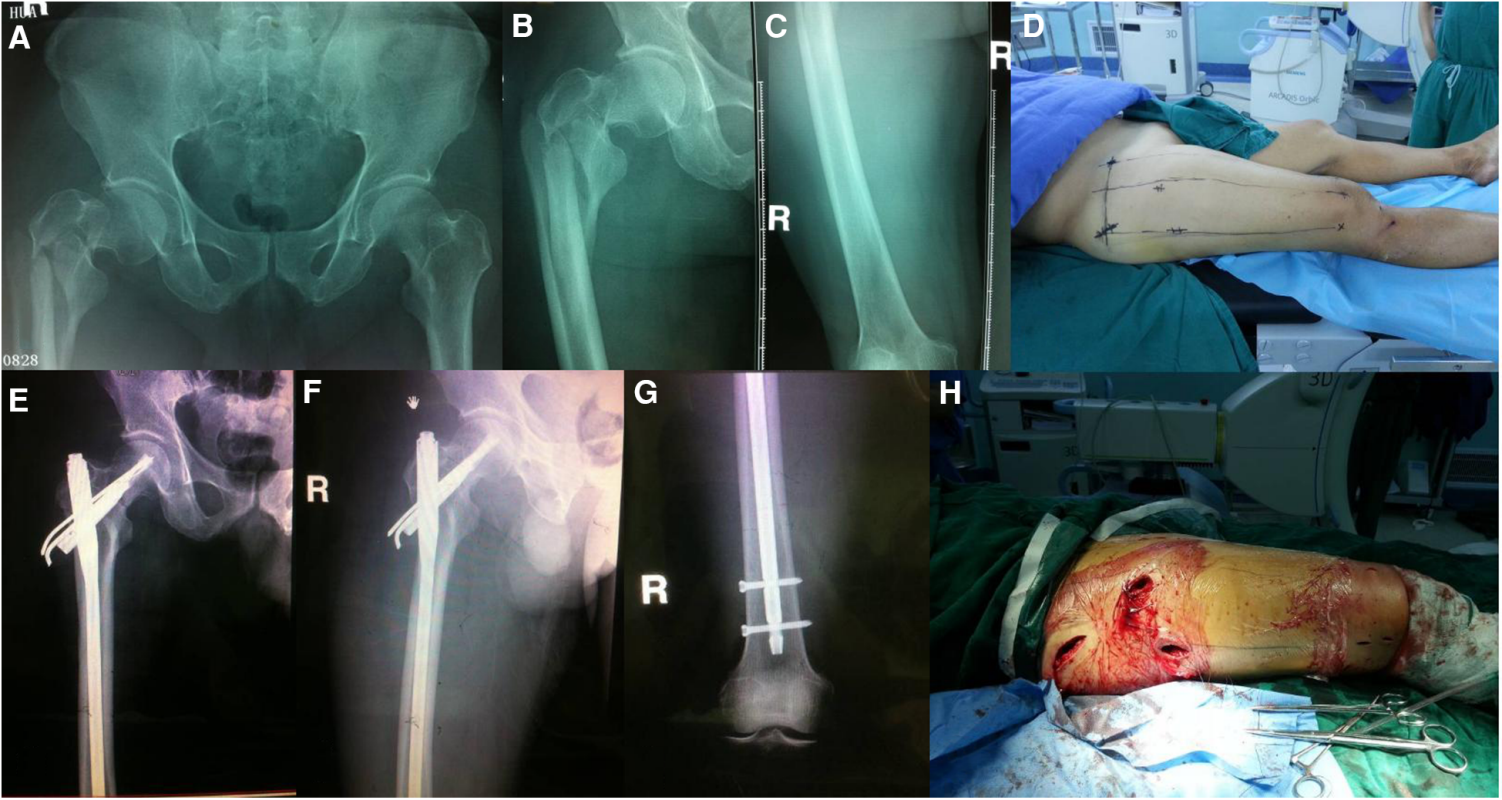
Preoperative anterolateral x-rays of the patient's hip joint **(A–C)** preoperative localization of skin incision was performed by the “3-2-1” body surface localization method **(D)** postoperative anterolateral x-rays of the patient's hip joint and left lower extremity showed good fracture reduction **(E–G)** the postoperative incision was consistent with the preoperative planning, achieving a minimally invasive “3-2-1" **(H)**.

Case 3 referred to a 62-year-old male patient who was admitted to the emergency department with “intertrochanteric fractures accompanied with subtrochanteric femoral and femoral neck fractures” causing traumatic right hip swelling and pain (Figures 11A–C). Surgical incisions were marked preoperatively using the “3-2-1” body surface localization method (Figure 11D). The patient underwent PFNA using a long intramedullary nail under general anesthesia, supine position, and non-traction bed conditions. Owing to the severe fracture damage, considering that short intramedullary nails could not achieve true stability, long intramedullary nails were used to increase stability. Therefore, during intraoperative fracture reduction, “needle insertion point incision, incision of the spiral blade, anterolateral auxiliary incision” was made to avoid opening the locking nail incision. Finally, the locking nail was inserted into the distal femur, and the procedure was completed. In multiple hip fractures, the fracture block is crushed, the soft tissue is incarcerated, and fracture reduction is difficult, requiring wide surgical incisions to reduce the difficulty of the operation. Our technique can avoid such an event. Gradual surgical reduction not only promotes surgical efficiency but also does not require wide incisions, even employing minimally invasive methods (Figures 11E–H).

## 5 Results

All patients underwent surgery using the body surface locator and lower limb force axis. This auxiliary positioning technique predicted the needle insertion point incision, spiral blade incision, and locking nail incision; achieved minimally invasive incision; avoided incorrect incision positioning; facilitated accurate intraoperative intramedullary nail placement; reduced the incision size, intraoperative bleeding, and radiation exposure; and greatly improved surgical efficiency and reduction quality.

## 6 Discussion

Intertrochanteric femoral fractures are more common in older people, accounting for 46.5% of the total hip fractures (1). Older patients with low physical activity and various underlying diseases have low hemoglobin levels because of massive bleeding caused by traumatic fractures (14). Studies have shown that hidden blood loss during the perioperative period of intertrochanteric femoral fractures accounts for 80% of the total blood loss, which is four times that of dominant blood loss (15). Hidden blood loss is associated with reaming of the marrow cavity and soft tissue injury (16). In our technique, we did not expand the marrow cavity during surgery and made minimally invasive incisions to shorten the operative time and reduce soft tissue injury, intraoperative bleeding, and postoperative complications. At present, intraoperative bleeding can be controlled by (1) making minimally invasive incisions to reduce intraoperative bleeding and promote surgical efficiency, (2) accurate positioning of the incision preoperatively to shorten the reduction time and reduce intraoperative bleeding, and (3) improving surgical proficiency and speed up surgical efficiency. In the third point, surgeon-related factors can be excluded. Although the use of minimally invasive incision can reduce intraoperative bleeding, the proximal fracture mass can be abducted, rotated, and flexed because of the traction of the abductor and rotator muscles around the femoral trochanter, and the distal fracture mass can be inverted and shortened because of the traction of adductor muscles (5). Therefore, reducing a fracture through a minimally invasive incision is difficult and sometimes counterproductive.

We summarized this in the field of preoperative localization of incisions. Lan et al. used orthopedic robots for preoperative positioning and navigation; although their results showed that robots can effectively shorten the operation time and reduce intraoperative bleeding, robots are not widely used in developing countries and are expensive, so they are not suitable for ordinary patients (6, 7). Jiang and Wu et al. used the body surface guide wire positioning method and the intervertebral mirror grid locator to locate the main nail entrance; however, they could not identify the location of the spiral blade incision (8, 9). Cheng et al. used the “three-finger method” to locate the proximal femur incision; however, the finger width of different operators varied to some extent, and positioning was subjective (10).

Considering the advantages and disadvantages of existing positioning methods, we developed our “3-2-1” body surface localization technique. By this technique, in fractures with mild displacement requiring simple intraoperative closed reduction, we use the combination of needle insertion point incision and spiral blade incision for reduction. For complicated fractures with displacement and intraoperative closed reduction is difficult (two-part intertrochanteric femoral fractures with bisection of the lesser trochanter, multiple hip fractures, etc.), three incisions (needle insertion point, spiral blade, anterolateral auxiliary incisions) were made in advance to achieve pulling, prying, jacking, or Kirschner wire temporary fixation of the fracture end to reduce the fracture end. This technique achieves a truly minimally invasive incision: (1) The needle insertion point incision is used to lift the gluteus media, assisting the distal side to resist traction, and realizing the preoperative fracture reduction. (2) The needle insertion point incision or spiral blade incision is used in conjunction with tools to reduce the fracture by prying. The anterolateral auxiliary incision is used for hook-pull reduction using bone hooks or vascular forceps in cases of internal and external inversion deformity where fracture reduction is difficult. Moreover, the spiral blade incision can be used in long oblique subtrochanteric fractures, and the steel wire circumferential ligature can be used to strengthen the fixation at the fracture site to promote fracture healing. The concept of the “3-2-1” body surface localization method is the “one-incision dual use” of the spiral blade incision that is, the reduction and placement of the spiral blade. During reduction, the spiral blade incision is used for “lateral unlock,” whereas the anterolateral auxiliary incision is used for “anterior unlock.” To achieve minimal invasiveness, reasonable preoperative incision planning reduces the difficulty of reduction. The minimally invasive incision in the “3-2-1” body surface localization method enables direct exposure of the fracture, which allows for identifying the displacement direction of the fracture end during surgery, avoiding the use of a C-arm x-ray machine for fluoroscopic positioning, greatly reducing radiation exposure to the surgeon, and protecting doctors and patients. It can also protect the blood supply of the fracture end as much as possible and the normal biological environment of the bone and promote fracture healing.

In the “3-2-1” body surface localization method, the surgical incision is marked through the lower limb force line using the body surface locator, which solves the subjectivity in positioning by past researchers. The technique accurately locates the incision site for the spiral blade and achieves “one-incision dual use,” which solves the reported shortcoming of the inability to locate the incision for the spiral blade. The technique is low cost, uses easy access to tools, and can be carried out in some underdeveloped settings, solving the shortcomings of the unpopularity of robot use. The technique is made possible using the lower limb force line and the body surface locator, which not only facilitate the precise insertion of the intramedullary nails during the operation but also avoid incorrect incision positioning. The incisions for the needle insertion point, spiral blade, and locking nails are predicted to reduce the number and size of incisions and achieve minimal invasiveness. Minimally invasive incisions allow for direct visualization of the fracture, facilitate intraoperative identification of the displacement direction of the fracture end, and reduce intraoperative radiation exposure. These three points improve surgical efficiency and quality. Moreover, this surgical technique has no obvious contraindications. However, in patients with very loose skin and obesity, the use of a body surface locator will affect the accurate positioning of the surgical incision. However, in patients with an intertrochanteric femoral fracture combined with a hip fracture, the presence of intertrochanteric femoral fractures combined with acetabulum fractures causing severe displacement of the anterior superior iliac spine disturbs the positioning of the anterolateral auxiliary incision axis and thus the use of the locator. The “3-2-1” body surface localization method is worthy of promotion in the orthopedics field; however, at present, this technique is only used in some patients, and the degree of fracture recovery needs to be studied. In addition, this technology has not been evaluated by randomized controlled trials using large samples; thus, our team will focus on this direction in the future.

## Data Availability

The datasets presented in this study can be found in online repositories. The names of the repository/repositories and accession number(s) can be found in the article/Supplementary Material.
